# MBNL1 regulates isoproterenol‐induced myocardial remodelling in vitro and in vivo

**DOI:** 10.1111/jcmm.16177

**Published:** 2020-12-08

**Authors:** Yao Xu, Chen Liang, Ying Luo, Tongcun Zhang

**Affiliations:** ^1^ College of Life Sciences and Health Wuhan University of Science and Technology Wuhan China; ^2^ College of Biological Science and Technology Hubei Minzu University Enshi China; ^3^ Hubei Provincial Key Laboratory of Occurrence and Intervention of Rheumatic diseases Hubei Minzu University Enshi China

**Keywords:** feedback loop, MBNL1, myocardial remodelling, Myocardin, post‐transcriptional regulation

## Abstract

Myocardial remodelling is a common phenomenon in cardiovascular diseases, which threaten human health and the quality of life. Due to the lack of effective early diagnosis and treatment methods, the molecular mechanism of myocardial remodelling should be explored in depth. In this study, we observed the high expression of MBNL1 in cardiac tissue and peripheral blood of an isoproterenol (ISO)‐induced cardiac hypertrophy mouse model. MBNL1 promoted ISO‐induced cardiac hypertrophy and fibrosis by stabilizing Myocardin mRNA in vivo and in vitro. Meanwhile, an increase in MBNL1 may induce the apoptosis of cardiomyocytes treated with ISO via TNF‐α signalling. Interestingly, MBNL1 can be activated by p300 in cardiomyocytes treated with ISO. At last, Myocardin can reverse activate the expression of MBNL1. These results suggest that MBNL1 may be a potential target for the early diagnosis and clinical treatment of myocardial remodelling.

## 1 INTRODUCTION

Myocardial remodelling is common in many cardiovascular diseases such as coronary atherosclerotic heart disease, hypertension, hypertrophic cardiomyopathy and heart failure and is due to the activation of multiple stimulating factors and intracellular signal transduction mechanisms.^1^ Chronic heart failure and heart failure are important links in the progression of various cardiovascular diseases. Cardiac remodelling mainly includes myocardial hypertrophy and interstitial fibrosis. The structure, metabolism and function of cardiac tissues change according to gene expression states. Moreover, cardiac morphology changes, cardiac hypertrophy, cardiomyocyte apoptosis and extracellular matrix network remodelling occur and lead to chronic heart failure, arrhythmia and eventually cardiogenic shock.

Myocardial hypertrophy plays an important role in cardiac remodelling.^2^, ^3^ It cannot only stimulate collagen synthesis and promote interstitial fibrosis but also induce the activation of matrix metalloproteinases (MMPs), which degrade collagen, and eventually lead to ventricular dilation. Therefore, blocking cardiac hypertrophy is a key to the prevention and treatment of cardiac remodelling. At the molecular level, hypertrophic stimuli exert effects on cell membrane effector molecules and sensitize signal transduction pathways that are related to cardiac hypertrophy in the cytoplasm and activate the transcription of hypertrophy‐related molecules^4^; therefore, the discovery of molecules that can inhibit or promote myocardial hypertrophy would be helpful in finding ways to block the occurrence and development of myocardial hypertrophy.

Myocardin consists of 35 amino acids arranged in a helix‐junction‐helix structure and is specifically expressed in embryonic, adult myocardium and vascular smooth muscle cells.^5^, ^6^, ^7^ Previous studies have confirmed that Myocardin can specifically bind the DNA sequence CarG box (CC (A/T) _6_G box) in the promoter region of target genes and activate the expression of the target genes by forming a complex with serum response factor (SRF), which also result in the regulation of the development, growth, differentiation, and apoptosis of heart and smooth muscle cells.^8^, ^9^, ^10^, ^11^, ^12^ In particular, the high expression of Myocardin is an important inducer of the occurrence and development of myocardial hypertrophy and has attracted wide attention^13^, ^14^, ^15^; therefore, elucidating the regulation mechanism of Myocardin expression is important.

RNA‐binding proteins (RBPs) are a class of proteins that directly bind to RNAs. As key participants in post‐transcriptional regulation, RBPs control RNA processing at multiple levels, including alternative splicing, RNA stability, RNA localization and translation efficiency.^16^, ^17^, ^18^, ^19^ The correlation of RBPs with cell differentiation and metabolic diseases has been confirmed.^20^, ^21^, ^22^ MBNL1, which belongs to the family of muscle blind‐like (MBNL) proteins, is closely linked to the occurrence and development of many diseases owing to their regulation of RNA metabolism in several ways, including splicing of alternative exons, selection of a polyadenylation site in pre‐mRNAs, influencing the stability and differential localization of mRNAs, and processing of miRNAs.^23^, ^24^, ^25^, ^26^, ^27^, ^28^ To date, the relationship between MBNL1 and myocardial hypertrophy remains unclear.

We used bioinformatics tools and found that Myocardin mRNA and the promoter region of MBNL1 contain multiple MBNL1 binding sites and a Myocardin‐binding site, respectively. Therefore, we speculate that there may be an interaction between MBNL1 and Myocardin. This article focuses on the interaction between MBNL1 and Myocardin and their underlying molecular mechanism in regulating myocardial hypertrophy.

## 2 MATERIALS AND METHODS

### 2.1 Ethical approval

In this study, all animal‐related experiments were approved by the Institutional Animal Care and Use Committee of the Tianyou Hospital Affiliated to Wuhan University of Science and Technology. And all animal experiments conformed to the ethical standards outlined in the Guide for the Care and Use of Laboratory Animals (NIH publication no. 85‐23, revised 1996).

### 2.2 Animal studies

Two‐month‐old wild‐type C57BL/6 mice were purchased from the Animal Experimental Centre of Hubei Academy of Preventive Medicine. All mice were fed in SPF level animal laboratory under controlled environmental conditions (12 hours light/dark cycle and room temperature 20 ± 5°C). And all mice were free to obtain standard laboratory food and water. Mice with different treatments were infused with ISO for 2 weeks and saline‐infused mice served as controls. After 14 days, mice were anaesthetised with 50 mg/kg pentobarbital sodium (Sigma) via intraperitoneal injection. No toe pinch reflex confirmed adequate anaesthesia. Then, the mice were killed by cervical dislocation, and hypertrophic analysis was performed.

### 2.3 Cardiomyocyte isolation and culture

Primary neonatal mouse cardiomyocytes were isolated from the heart of 2‐ to 3‐day‐old C57BL/6 mice with mild enzymatic (0.1% collagenase type II and 0.08% trypsin) digestion and gentle mechanical attrition in sterile environment. After centrifugation, cells were incubated in DMEM/F‐12 (Gibco.) containing 10% foetal bovine serum (Gibco.), 100 U/mL penicillin, 100 µg/mL streptomycin and 0.1 mmol/L bromodeoxyuridine.

### 2.4 Cell culture

The Cos‐7 (CRL‐1651) and HEK 293T/17 (CRL‐11268) cells used in this study were purchased from ATCC. The cells were seeded in High Glucose DMEM (Hyclone) supplemented with 10% FBS (Gibco.), penicillin (100 U/mL) and streptomycin (100 U/mL) at 37°C in humidified air with 5% CO_2_.

### 2.5 Standard lentivirus production

MBNL1 was inserted into a lentivirus vector pCDH‐αMHC. The plasmids pCDH‐αMHC‐MBNL1 or pCDH‐αMHC (as control) were cotransfected with psPAX2 and pMG2.G into 293T cells (approximately 70%‐80% confluency). After 8 hours of transfection, the medium was replaced, and then, supernatant was filtered after 48 hours to obtain active lentivirus.

### 2.6 Lentivirus shRNA plasmids construction

The target shRNAs against mouse MBNL1, Myocardin and p300 were inserted into pαMHC‐clone26. A negative control showing no significant homology to any mouse or human gene was inserted into pαMHC‐clone26. ShRNAs lentivirus were produced by cotransfecting 293T cells with the lentivirus expression plasmid and packaging plasmid. Interference efficiency was measured by realtime PCR and Western blotting.

### 2.7 Lentivirus transduction of primary mouse cardiomyocyte

Lentivirus was added to primary mouse cardiomyocyte cultured in low glucose DMEM supplemented with 10% FBS at a ratio of 50 multiplicity of infection (MOI). After overnight incubation, fresh medium was replaced.

### 2.8 Quantitative realtime PCR (qRT‐PCR)

According to the manufacturer's protocol, the total RNA was extracted by using the Total RNA Extraction Kit (OMEGA). Then, the total RNA extracted was reverse transcribed using M‐MLV Reverse Transcriptase (Promega). Finally, realtime PCR was performed in a StepOne Realtime PCR System (Thermo) with Fast SYBR Green Master Mix (Thermo). The relative expression levels of related genes were normalized to GAPDH. The primers for the realtime PCR analysis are as follows: MBNL1, F: 5′‐CTTGCTCACGACCAGAC‐3′, R: 5′‐ATTCCGCCCATTTATC‐3′; Myocardin, F: 5′‐CTCAACCCTTGTCCCA‐3′, R: 5′‐GTCATTTGCTGCTTCACT‐3′; ACTN2, F: 5′‐AAACCCGATGAAAGAGC‐3′, R: 5′‐GCCAGGGGATTGTGC‐3′; ANP, F: 5′‐GGGCTTCTTCCTCGTC‐3′, R: 5′‐TCTCCTCCAGGTGGTCTA‐3′; p300, F: 5′‐TGAGGATACAAAGGAGGCTA‐3′, R: 5′‐CTGACGAAAGGGAAGAGATT‐3′; TNF‐α, F: 5′‐TGTCTACTGAACTTCGGGGT‐3′, R: 5′‐ TTGGTGGTTTGTGAGT GTGA‐3′; GAPDH, F: 5′‐CAGTGCGGTGTCCAAC‐3′, R: 5′‐GACCTCCCCAGT CCAG‐3′.

### 2.9 Protein extraction and Western blotting

For Western blotting analysis, protein samples were extracted from the cells through protein extraction reagent (Thermo) and the concentration of protein samples was determined by BCA Quantitative Kit (Beyotime). Then, the difference of protein expression was analysed by Western blotting analysis. The antibodies used in this process were as follows: rabbit anti‐Myocardin (DF2434, Affinity), rabbit anti‐MBNL1 (ab45899, Abcam), mouse anti‐ACTN2 (ab9465, Abcam), rabbit anti‐ANP (ab180649, Abcam), rabbit anti‐TNF‐α (#3707, CST), rabbit anti‐p300 (#86377, CST) and rabbit anti‐GAPDH (#97166, CST). GAPDH expression was used as an internal control.

### 2.10 Luciferase constructs and luciferase assay

The wild type and mutant of MBNL1 promoter were inserted into pGL3 and amplified with primers as follow: F1: GGCGACGCGTGTAGGCTAAGCTAGCCTT GGG, F2: GCGGACGCGTTCACATTCCATCCAGTTGCT, R: TATACTCGAGGC CCCTCCCCCGCGCCA. Cos‐7 cells (2 × 10^5^/well, 24‐well plates) were cotransfected with pcDNA3.1 (as control) or Myocardin in combination with pGL3‐MBNL1‐WT or pGL3‐MBNL1‐MUT. The Dual Luciferase Assay Kit (Promega) was used to measure the difference of luciferase activity in each experimental group.

### 2.11 Immunofluorescence analysis

The treated cells were immobilized with 4% paraformaldehyde for 15 minutes, washed with PBS and blocked with normal goat serum for 20 minutes at room temperature. Then incubated with Rhodamine Phalloidin (R415, Thermo) for 15 minutes and washed with PBS, the samples were observed by laser scanning confocal microscope (OLYMPUS).

### 2.12 RNA pulldown

In vitro, Myocardin mRNA and TNF‐α mRNA were transcribed by T7 RNA polymerase (Sigma) and the RNA (Roche) was labelled with the Biotin RNA Labeling Mix (Roche). Whole‐cell lysates (1 mg) of primary mouse cardiomyocytes were then incubated with 3 µg of purified biotinylated transcripts for 1 hour at 25°C. Proteins bound to transcripts in pull‐down materials were detected by streptavidin agarose beads (Invitrogen) combined with Western blotting.

### 2.13 RNA immunoprecipitation (RIP)

2 × 10^7^ primary mouse cardiomyocytes were taken to carry out RIP experiments combined with anti‐IgG or anti‐MBNL1. The RNA in the coprecipitation was then extracted and detected by realtime PCR. The gene‐specific primer used to detect Myocardin is as follows: F: 5′‐CTCAACCCTTGTCCCA‐3′, R: 5′‐GTCATTTGCTGCTTCAC T‐3′.

### 2.14 Histological analysis (H&E, WGA, Masson's Trichrome staining)

The excised mouse hearts were fixed with formaldehyde at 4°C for 72 hours and embedded in paraffin. Then, the paraffin‐embedded tissues were cut into 3‐4 µm slices. The sections were then dewaxed with xylene for 15 minutes, rehydrated in gradient alcohols, and stained with haematoxylin‐eosin (H&E) or wheat germ agglutinin (WGA) or Masson trichrome. Five random fields were selected for capture under an inverted microscope (Olympus, Tokyo, Japan).

### 2.15 TUNEL assay

After different lentiviral transductions, apoptotic cells in each well (24‐well plates) or in each section (3‐4 µm slices) were visualized using TUNEL according to the manufacturer's instructions (Promega). The experimental results of TUNEL assay were observed under a confocal microscope (OLYMPUS F3000).

### 2.16 TNFα secretion assays

Enzyme‐linked immunosorbent assay (ELISA, BMS607‐3, Invitrogen) was used to detect the secretion of TNF‐α in cell cultures according to the manufacturer's instructions. Briefly, supernatant of primary cardiomyocytes with different treatment was collected and centrifuged (500 *g*) for 5 minutes. Then, the supernatant was subjected to ELISA assay.

### 2.17 Chromatin immunoprecipitation (ChIP) assay

We performed chromatin immunoprecipitation assay by using a commercial chip assay kit (#56383, CST) according to the manufacturer's instructions. Briefly, each group to be tested was incubated with 1% formaldehyde to cross‐link DNA‐protein complexes. Cells were then collected, washed three times with ice‐cold PBS and lysed in SDS lysis buffer. After centrifugation, the lysates were ultrasonically treated and the DNA was cut into 200‐1000 bp fragments. We then immunoprecipitate the cross‐linked protein at 4°C overnight using an anti‐Myocardin antibody (DF2434, Affinity). IgG acted as the negative control. Finally, DNA from the coprecipitated material was extracted and used as a template for PCR to find the binding site of Myocardin. The primers for PCR analysis are shown below: F: 5′‐CTGCATGAGTCAGTTTTCCA‐3′, R: 5′‐ATT AACT TGTCGGCAGAGAAG‐3′.

### 2.18 Trichostatin A (TSA) treatment

In this study, we chose TSA (Sigma) as an inhibitor of p300. We treated primary cardiomyocytes with 100 ng/mL TSA for 24 hours. Then, cells were replaced the normal medium and carried out ISO induction. After ISO induction, proteins were collected from cells for Western blotting analysis.

### 2.19 Measurement of RNA stability

Primary cardiomyocytes were transduced with MBNL1 or sh‐MBNL1 lentivirus. Transcription was stopped using Actinomycin D (ACD, 5 µg/mL), and RNAs were extracted at various time points (0, 20, 40 and 60 minutes) following Actinomycin D treatment. Realtime PCR assay was detected the remaining of Myocardin mRNA and adjusted by GAPDH levels. The primer for the realtime PCR analysis is as follows: Myocardin, F: 5’‐CTCAACCCTTGTCCCA‐3’, R: 5’‐GTCATTTGCTGCTTCACT‐3’. The corrected density was then plotted as a percentage of different time points value against control, with the decay rate constant derived from the slope of the decay curve.

### 2.20 Statistical analysis

High throughput sequencing data were obtained from the GEO database (GSE129090). Quantitative data are expressed as mean ± SEM. Statistical analysis of differences between two groups was performed by Student's *t* test. A one‐way analysis of variance followed by Tukey test was performed to compare differences among multiple groups. Statistical analysis was performed with GraphPad Prism 8. *P* < .05 indicated statistically significant difference.

## 3 RESULTS

### 3.1 MBNL1 is highly expressed in myocardial hypertrophy mouse model

**FIGURE 1:**
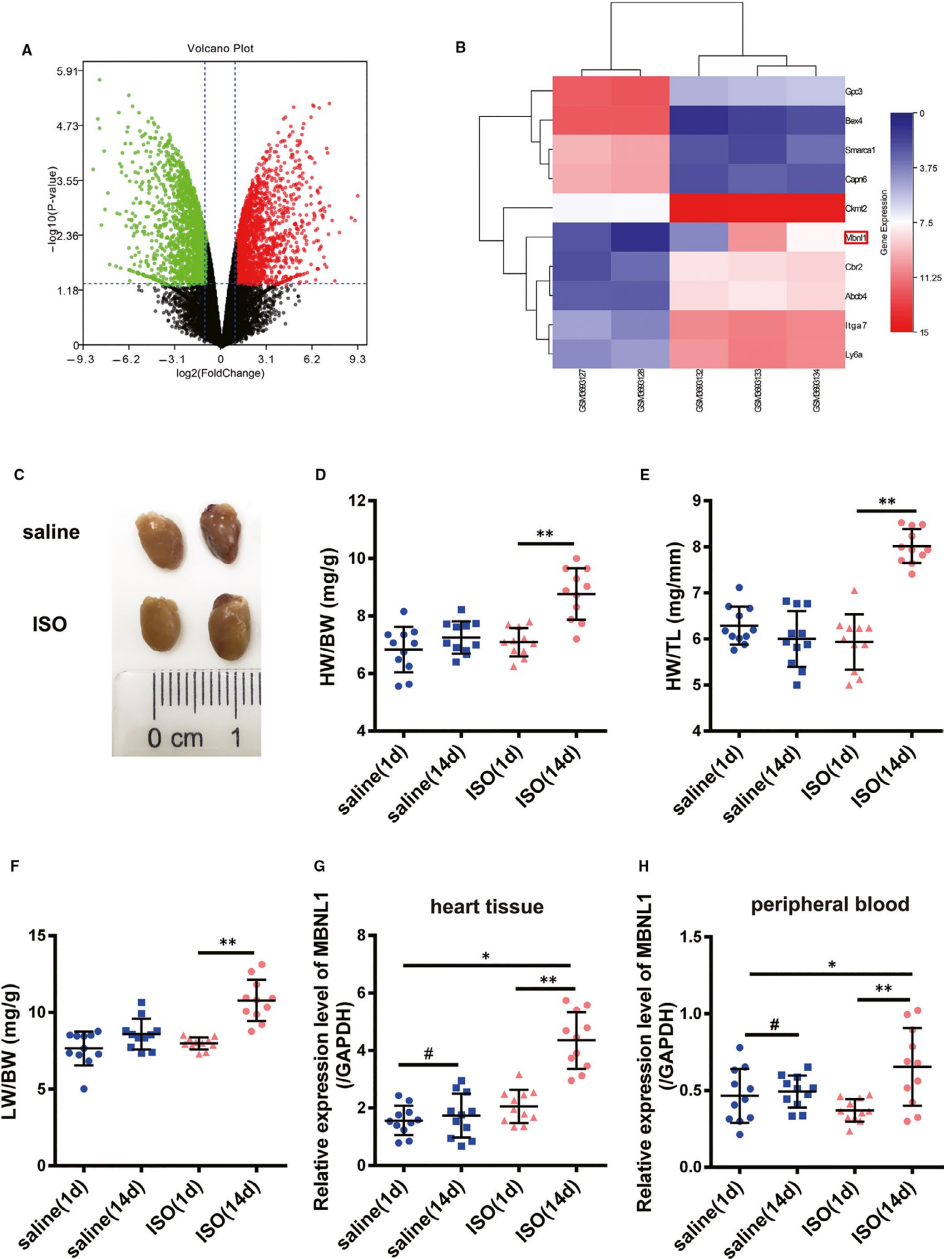
MBNL1 is highly expressed in myocardial hypertrophy mouse model. A and B, Difference in MBNL1 expression in the heart of hypertrophic mice and normal mice is shown in the volcano plot and heat map. C, Gross hearts under natural light after treatment with ISO. Scale bars represent 2 mm. D, E, and F, Ratio of heart weight to bodyweight (HW/BW), heart weight to tibia length (HW/TL) and lung weight to bodyweight (LW/BW) in different groups (Each group n = 11, **, *P* < .01). G and H, mRNA levels of MBNL1 in the heart or peripheral blood of mice after intraperitoneal injection with ISO or saline (Each group n = 11, *, *P* < .05, **, *P* < .01, ^#^, *P* > .05)

By analysing the data in the GEO database (GSE129090), we found that MBNL1 was highly expressed myocardial hypertrophy samples (Figure 1A,B). To verify the above results, a cardiac hypertrophy mouse model was induced using ISO (Figure 1C‐F). MBNL1 expression in mouse cardiomyocytes was detected using realtime PCR. As shown in Figure 1G, the level of MBNL1 was significantly higher in myocardial hypertrophy mice than in normal mice. Interestingly, the level of MBNL1 in the peripheral blood of mice treated with ISO was also significantly higher than that in the control group (Figure 1H). This suggests that MBNL1 may be a biomarker of myocardial remodelling.

### 3.2 The overexpression of MBNL1 contributes to ISO‐induced myocardial remodelling in vivo and in vitro

**FIGURE 2:**
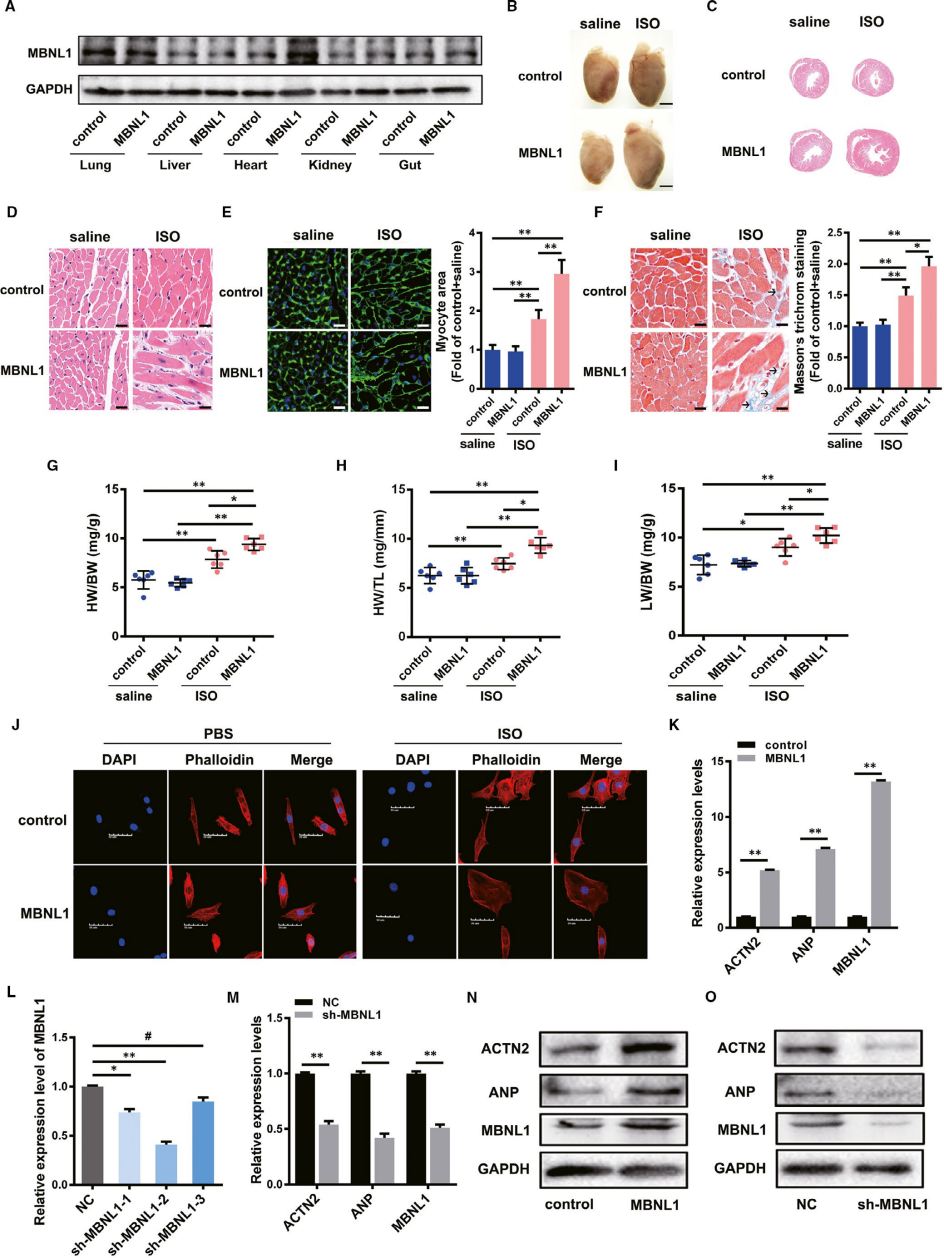
The overexpression of MBNL1 contributes to ISO‐induced myocardial remodelling in vivo and in vitro. A, Detection of MBNL1 overexpression effects. B, Gross hearts under natural light. Scale bars represent 2 mm. C, Representative images of the cross‐sections of ventricles stained with H&E (20× magnification). D, 400× microscopic views of H&E sections. Scale bars represent 50 µm. E and F, The myocyte areas and cardiac fibrosis areas were measured using wheat germ agglutinin (WGA) staining and Masson's trichome staining, respectively. Scale bars represent 50 µm; each group n = 6, *, *P* < .05, **, *P* < .01. G, H and I, Ratio of heart weight to bodyweight (HW/BW), heart weight to tibia length (HW/TL) and lung weight to bodyweight (LW/BW) in different groups (Each group n = 6, *, *P* < .05, **, *P* < .01). J, The surface areas of cardiac myocytes after treatment with saline or ISO were determined using immunostaining and confocal microscopy. The cytoskeleton was stained with phalloidin, and the nuclei were counterstained with 4′,6‐diamidino‐2‐phenylindole (DAPI). Scale bars represent 50 µm. L, Detection of the effect of shRNA against MBNL1 in primary cardiomyocytes. (n = 3, *, *P* < .05, **, *P* < .01, #, *P* > .05). K, M, N and O, Primary cardiomyocytes were transduced with MBNL1 or sh‐MBNL1 lentivirus. The changes in ACTN2, ANP and MBNL1 were detected using realtime PCR and Western blotting. (n = 3, **, *P* < .01)

To investigate the potential effect of MBNL1 on cardiac remodelling, a lentivirus was used to produce a cardiac‐specific MBNL1 overexpression C57BL/6 mouse model. The cardiac MBNL1 protein in each group of mice was confirmed by using Western blotting (Figure 2A). On this basis, we performed intraperitoneal injection of ISO in half of the mice in each group, and the other half was injected with saline as a control. H&E and WGA staining techniques were used to analyse the heart tissue sections of mice in each group. Significantly increased cross‐sectional areas of the heart of MBNL1 mice were observed (Figure 2B‐E). Moreover, as shown in Figure 2G‐I, the heart weight to bodyweight ratios (HW/BW), heart weight to tibia length ratios (HW/TL) and lung weight to bodyweight ratios (LW/BW) in the MBNL1 group, after injection with ISO for two weeks, were all significantly increased compared with those in the control. Notably, increased fibrosis induced by ISO was observed in the hearts of mice with overexpressed MBNL1 compared with those of normal mice treated with ISO (Figure 2F). These results suggest that MBNL1 promotes myocardial hypertrophy in vivo.

To explore the function of MBNL1 in vitro, we used lentivirus to overexpress or silenced MBNL1 in primary cardiomyocytes. As a result, a significantly increased cross‐sectional area of the primary myocardium after treatment with ISO, in the context of MBNL1 expression and compared with that of the control, was observed by myocardial cytoskeleton staining with phalloidin (Figure 2J). Then, the changes in the expression of two cardiac hypertrophy‐specific genes, namely ACTN2 and ANP, in the primary myocardium with overexpressed or silenced MBNL1 were detected using realtime PCR and Western blotting. We observed that ACTN2 and ANP expression increased or significantly decreased compared with the control group, whether at the mRNA or protein level in overexpressed or silenced MBNL1 (Figure 2K‐O). These data preliminarily confirmed that MBNL1 can promote ISO‐induced cardiac hypertrophy in vitro.

### 3.3 MBNL1 increases Myocardin expression by binding to UGCU at the 3'‐UTR of Myocardin mRNA

**FIGURE 3:**
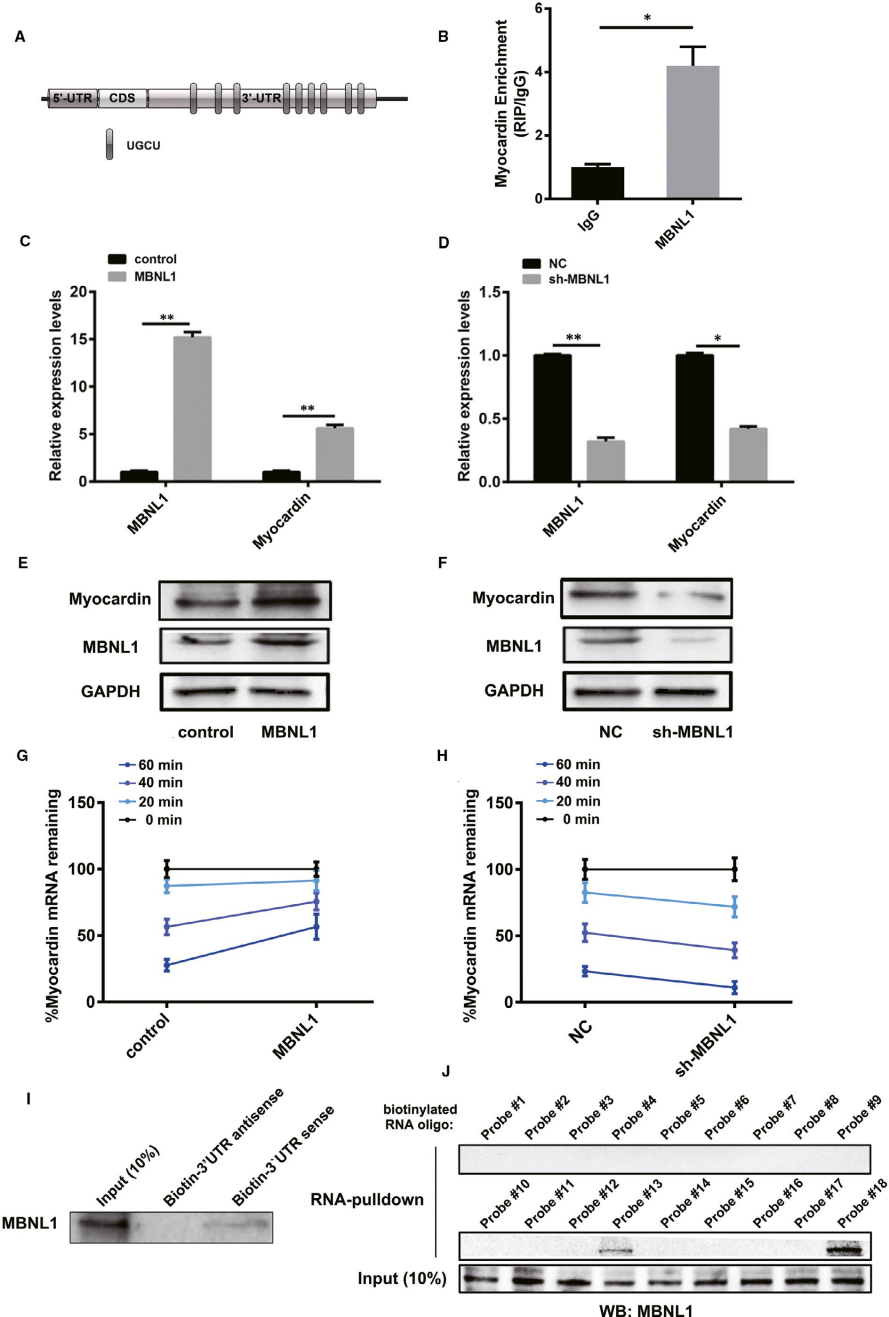
MBNL1 increases Myocardin expression by binding to UGCU at the 3'‐UTR of Myocardin mRNA. A, The 3'‐UTR region of Myocardin contains the binding site of MBNL1. B, The binding of MBNL1 to Myocardin mRNA was validated using RIP. (n = 3, *, *P* < .05). C‐F, The changes in Myocardin and MBNL1 in cardiomyocytes with overexpressed or silenced MBNL1 were measured using realtime PCR and Western blotting. (n = 3, *, *P* < .05, **, *P* < .01). G and H, Myocardin mRNA residues after 0, 20, 40 and 60 min in cardiomyocytes treated with Actinomycin D (ACD, 5 μg/mL) and with overexpressed or silenced MBNL1were measured using realtime PCR. I, The 3'‐UTR region of Myocardin mRNA was transcribed in vitro and labelled with biotin. The combination of MBNL1 protein and 3'‐UTR of Myocardin was detected using RNA pulldown assay. J, The RNA probes corresponding to nine prediction sites (probe # 10‐18) and their corresponding antisense probes (probe # 1‐9) were synthesized and biotin‐labelled. The combination site of MBNL1 protein in the 3'‐UTR of Myocardin was confirmed using RNA pull‐down assay

As an RNA‐binding protein, MBNL1 can regulate the stability of the target mRNA by binding to the 3'‐UTR region, thereby regulating the expression of the target gene. Interestingly, the 3'‐UTR of Myocardin mRNA contained multiple binding sites of MBNL1 (UGCU), suggesting that Myocardin may be a potential target for MBNL1 (Figure 3A). The RNA immunoprecipitation (RIP) assay confirmed that MBNL1 protein can bind to Myocardin mRNA (Figure 3B). Further realtime PCR and Western blotting results confirmed that MBNL1 can significantly up‐regulate the expression of Myocardin at both the mRNA and protein levels (Figure 3C‐F). Next, we examined the effect of MBNL1 on Myocardin mRNA degradation. The data indicated that MBNL1 overexpression significantly decreased the degradation rate of Myocardin mRNA (Figure 3G). The degradation rate of Myocardin mRNA increased significantly with silenced MBNL1 (Figure 3H). To confirm the binding site of MBNL1, the 3'‐UTR of Myocardin mRNA was reverse transcribed in vitro and labelled with biotin. As shown in Figure 3I, MBNL1 protein could bind to the 3'‐UTR region of Myocardin mRNA. Furthermore, MBNL1 could bind to the fourth and ninth UGCU sites, which was confirmed by performing RNA pulldown with biotin‐modified RNA probes corresponding to each potential site (Figure 3J). These data confirmed that MBNL1 protein prevented Myocardin mRNA from degradation by binding to UGCU in the 3'‐UTR region, resulting in the up‐regulation of the expression of Myocardin.

### 3.4 MBNL1 regulates myocardial hypertrophy and fibrosis via Myocardin in vivo and in vitro

**FIGURE 4:**
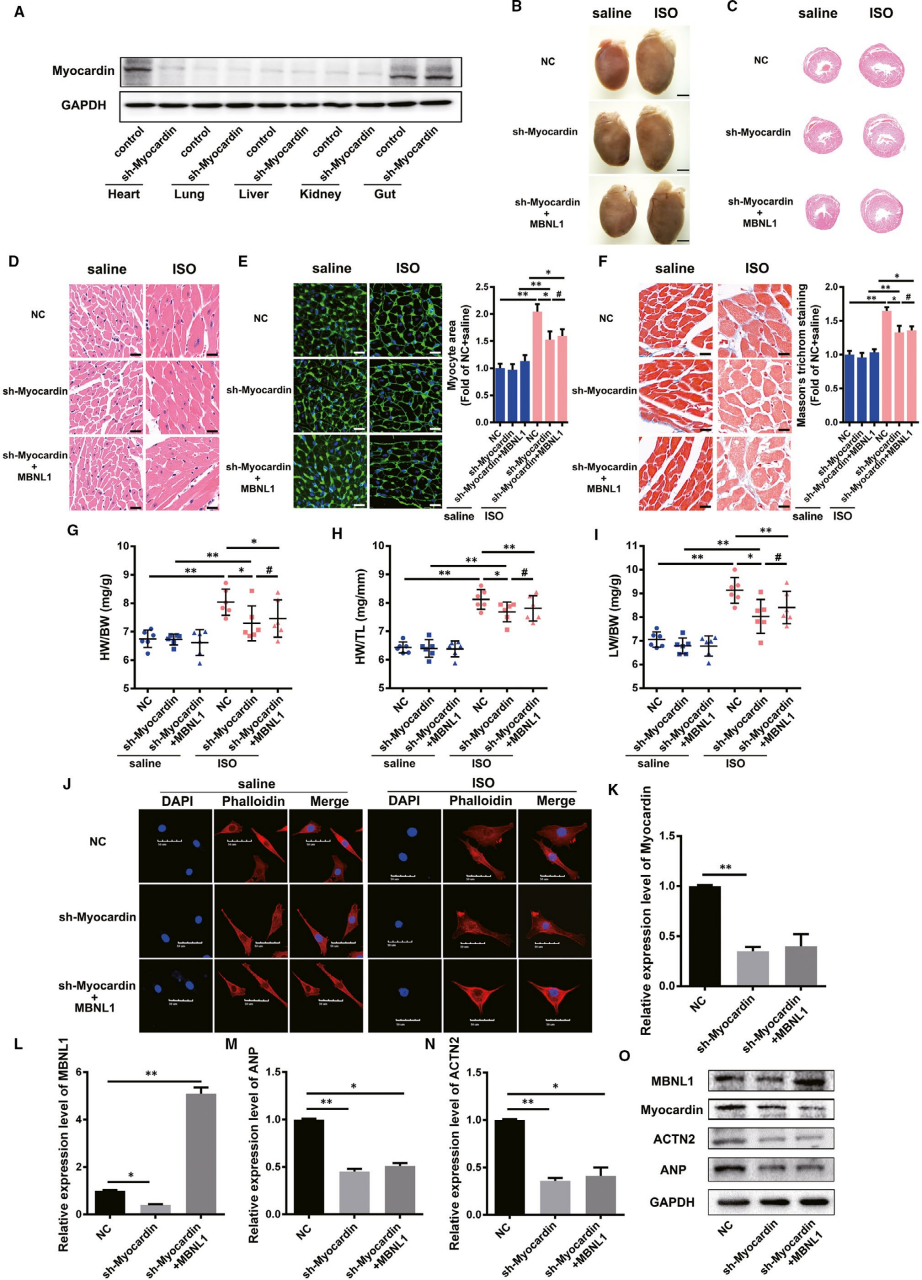
MBNL1 regulates myocardial hypertrophy and fibrosis via Myocardin in vivo and in vitro. A, Detection of Myocardin knock down effects in different tissues of mice. B, Gross hearts under natural light. Scale bars represent 2 mm. C, Representative images of cross‐sections of the ventricles stained with H&E (20 × magnification). D, 400× microscopic views of H&E sections. E and F, The myocyte areas and cardiac fibrosis areas were measured using WGA staining and Masson's trichome staining, respectively. Scale bars represent 50 µm, each group n = 6, *, *P* < .05, **, *P* < .01, ^#^, *P* > .05. G, H and I, Ratio of heart weight to bodyweight (HW/BW), heart weight to tibia length (HW/TL) and lung weight to bodyweight (LW/BW) in different groups (Each group n = 6, *, *P* < .05, **, *P* < .01, ^#^, *P* > .05). J, The surface areas of cardiac myocytes after treated with saline or ISO were determined using immunostaining and confocal microscopy. The cytoskeleton was stained with phalloidin, and the nuclei were counterstained with DAPI. Scale bars represent 50 µm. K‐O, Primary cardiomyocytes with Myocardin knock down were transduced with MBNL1 or sh‐MBNL1. The changes in ACTN2, ANP, Myocardin and MBNL1 were detected using realtime PCR and Western blotting (n = 3, *, *P* < .05, **, *P* < .01)

MBNL1 can regulate myocardial hypertrophy; Myocardin expression was previously confirmed. Here, we explored whether MBNL1 can regulate myocardial hypertrophy via Mycoardin using Myocardin‐knockdown (sh‐Myocardin) mice. First, the loss of Myocardin protein in the hearts of mice was confirmed by using Western blotting (Figure 4A). We overexpressed MBNL1 and silenced Myocardin, and all mice were divided into three groups: NC (negative control), sh‐Myocardin and sh‐Myocardin + MBNL1. Half of the mice in each group were injected with ISO, and the other half were injected with saline as a control. There was no difference between the cross‐sectional areas of cardiomyocytes of sh‐Myocardin and sh‐Myocardin + MBNL1 mice (Figure 4B‐E). Meanwhile, there was no significant difference between the fibrosis of the sh‐Myocardin and sh‐Myocardin + MBNL1 mice (Figure 4F). The heart weight to bodyweight ratios (HW/BW), heart weight to tibia length ratios (HW/TL) and lung weight to bodyweight ratios (LW/BW) were also measured in each group, and no significant difference in HW/BW, HW/TL and LW/BW ratios among sh‐Myocardin and sh‐Myocardin + MBNL1 groups was identified (Figure 4G‐I). This indicates that MBNL1 has no effect on cardiac hypertrophy during the lack of Myocardin.

Primary cardiomyocytes were used to verify the results of the above experiments. Phalloidin staining confirmed that MBNL1 could not effectively induce myocardial hypertrophy with Myocardin knockdown (Figure 4J). Similarly, realtime PCR and Western blotting results also confirmed that MBNL1 could not effectively activate the expression of ACTN2 and ANP expression in primary cardiomyocytes with silenced Myocardin, either at the mRNA or protein level (Figure 4K‐O).

### 3.5 MBNL1 may regulate ISO‐induced cardiomyocyte apoptosis via TNF‐α

**FIGURE 5:**
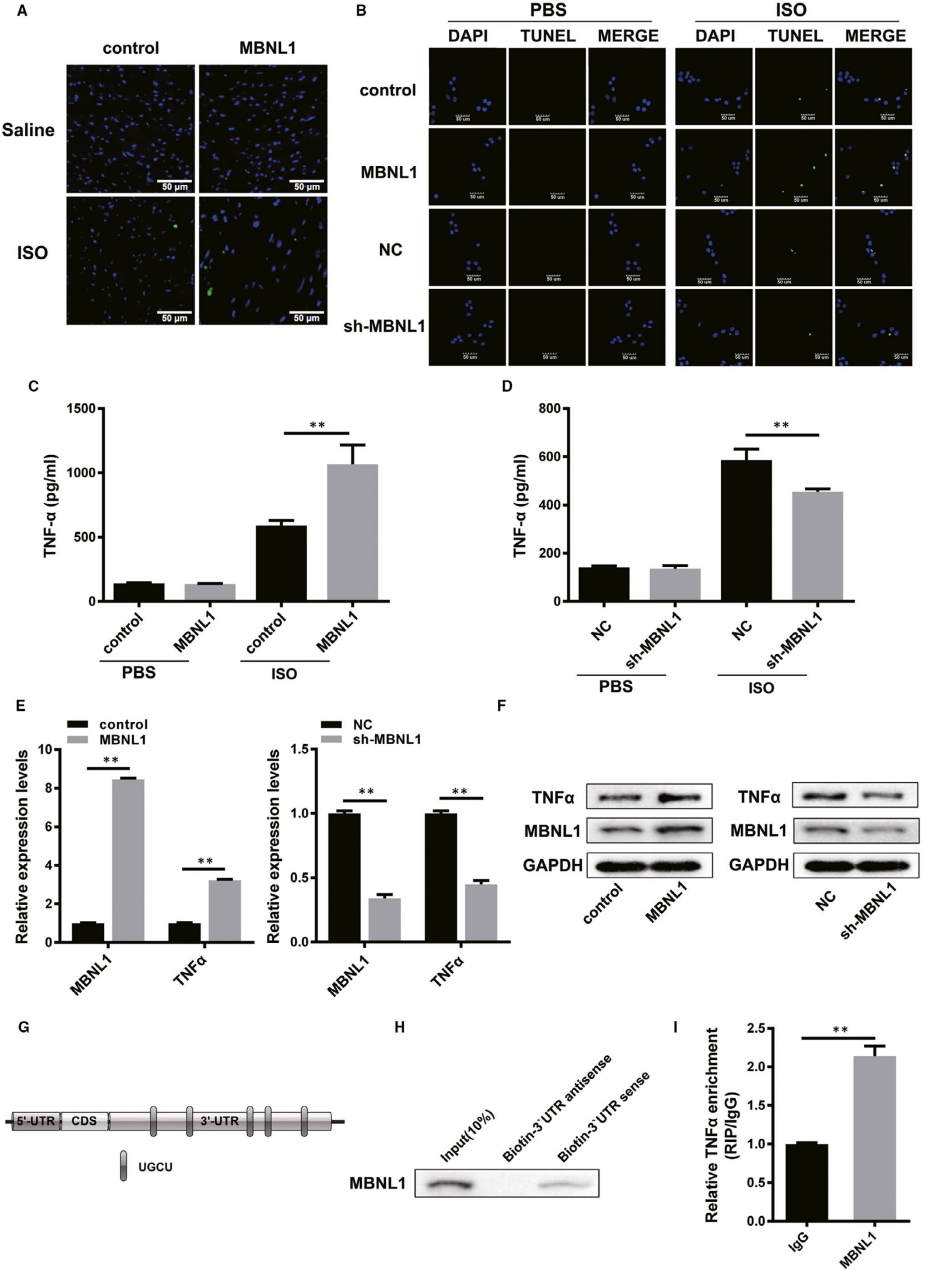
MBNL1 may regulate ISO‐induced cardiomyocyte apoptosis via TNF‐α. A, The apoptosis of cardiomyocytes in mice with overexpressed MBNL1 compared with normal mice treated with ISO was measured using TUNEL assay. Scale bars represent 50 µm. B, The apoptosis of cardiomyocytes after different treatments was detected using TUNEL assay under phosphate‐buffered saline (PBS) and ISO conditions. Scale bars represent 50 µm. C and D, The secretion of TNF‐α in primary cardiomyocytes with overexpressed or silenced MBNL1 after treatment with ISO or PBS was measured using ELISA. (n = 3, **, *P* < .01). E and F, The expression levels of TNF‐α in different groups of primary cardiomyocytes were measured using realtime PCR and Western blotting. (n = 3, **, *P* < .01). G, The 3'‐UTR region of TNF‐α contains the binding site of MBNL1. H and I, The combination site of MBNL1 protein in the 3'‐UTR of TNF‐α was confirmed using RNA pulldown and RIP assay. (n = 3, **, *P* < .01)

Excessive cardiac hypertrophy or myocardial fibrosis leads to cardiomyocyte apoptosis. In this part, we wanted to identify the effect of MBNL1 on cardiomyocyte apoptosis in ISO‐induced myocardial remodelling. As shown in Figure 5A, the overexpression of MBNL1 significantly increased the apoptosis rate of cardiomyocytes treated with ISO in vivo. Furthermore, the results of the terminal deoxynucleotidyl transferase dUTP nick end labeling (TUNEL) assay confirmed the above results in ISO‐induced primary cardiomyocytes (Figure 5B). In various acute and chronic diseases, apoptosis is often caused by an increase in free radicals and inflammatory factors (TNF‐α, IL‐6, etc). Consistently, we found that TNF‐α secretion significantly increased in the culture medium of primary cardiomyocytes with overexpressed MBNL1 treated with ISO (Figure 5C); however, the opposite result was obtained from cells with silenced MBNL1 and ISO treatment (Figure 5D). Meanwhile, the expression of TNF‐α was positively correlated with MBNL1 at both the RNA and protein levels (Figure 5E,F). Through bioinformatics analysis and prediction, we found a binding site of MBNL1 in the 3'‐UTR of TNF‐α mRNA (Figure 5G). Moreover, RNA pulldown and RIP assays confirmed that MBNL1 can bind to the 3'‐UTR of TNF‐α mRNA (Figure 5H,I). These data reveal that MBNL1 may regulate ISO‐induced cardiomyocyte apoptosis via TNF‐α.

### 3.6 The activation of MAPK and JNK signalling pathways can up‐regulate the expression of MBNL1 via p300 signalling in myocardial remodelling

**FIGURE 6:**
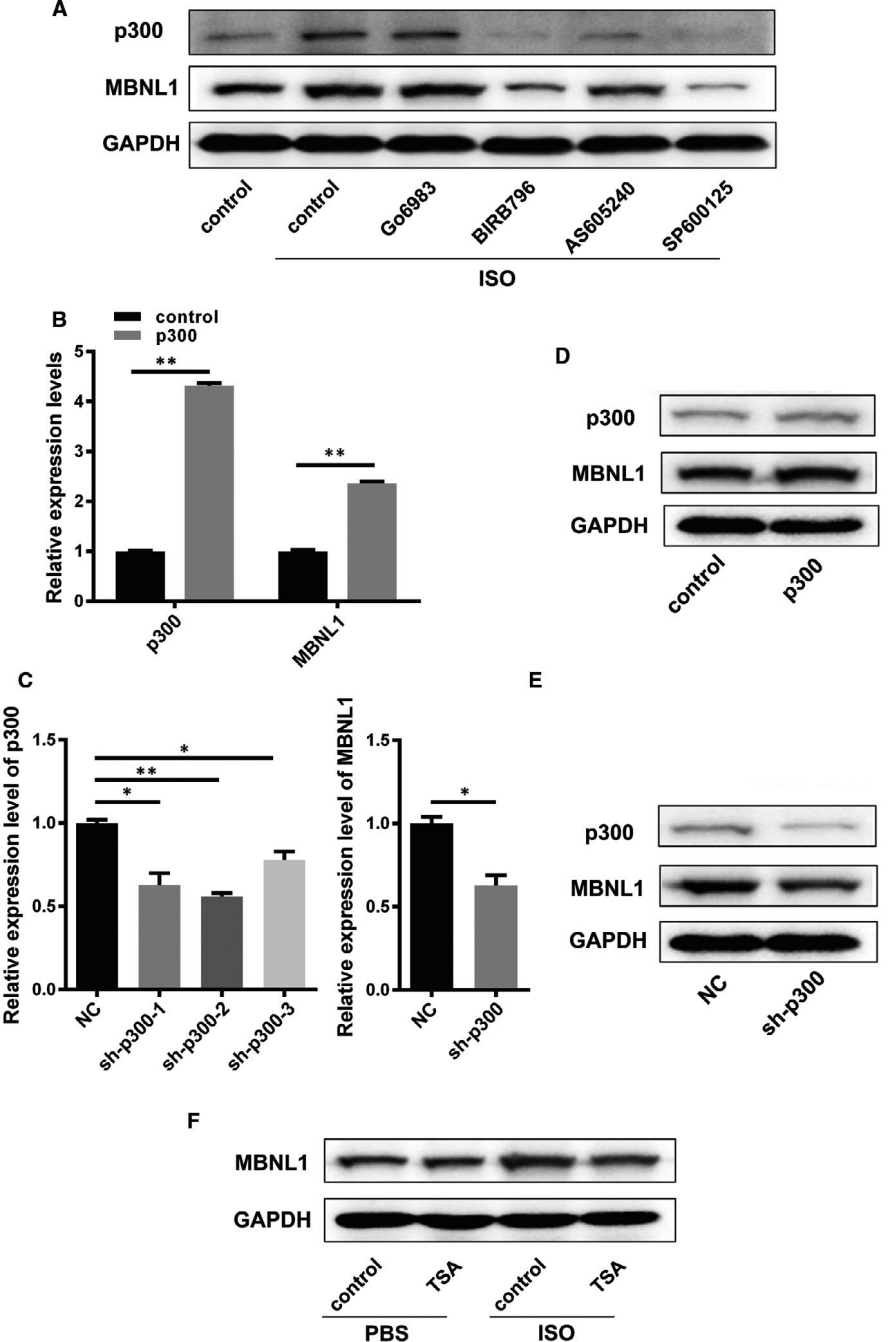
The activation of MAPK and JNK signalling pathways can result in the up‐regulation the expression of MBNL1 via p300 signalling in myocardial remodelling. A, Go6983, BIRB796, AS605240 and SP600125 were used to block AMPK, JNK, PKC and PI‐3K signalling pathways, respectively, in primary cardiomyocytes. The expression levels of MBNL1 in the above four groups in primary cardiomyocytes treated with ISO were measured using Western blotting. B‐E, The expression levels of MBNL1 in primary cardiomyocytes with overexpressed or silenced p300 were measured using realtime PCR and Western blotting. *(*n = 3, *, *P* < .05, **, *P* < .01). F, The function of p300 was blocked using TSA, and the expression levels of MBNL1 in primary cardiomyocytes treated with ISO or PBS were measured using Western blotting

We have revealed the function and molecular mechanism of MBNL1 in regulating myocardial remodelling; however, it is unclear how MBNL1 is activated during myocardial remodelling. Therefore, we tried to explore which signalling pathways, related to myocardial remodelling, are involved in MBNL1 activation. First, ISO was used to induce primary cardiomyocytes after treatment with inhibitors of MAPK, JNK, PI‐3K or PKC signalling pathways. As a result, the expression of MBNL1 was inhibited in ISO‐induced primary cardiomyocytes with blocked MAPK or JNK pathways (Figure 6A). In other words, the activation of MAPK and JNK can jointly regulate the expression of MBNL1. Subsequently, we analysed the important proteins associated with myocardial remodelling and can be regulated by MAPK or JNK signalling pathways. P300, as a histone acetyltransferase, can activate the expression of downstream target genes and be regulated by both MAPK and JNK, which may be key factors in the regulation MBNL1 (Figure 6A). To test this hypothesis, realtime PCR and Western blotting were used to determine the effect of p300 on MBNL1 expression. In primary cardiomyocytes, the overexpression of p300 resulted in a significant up‐regulation of MBNL1; however, the silencing of p300 can inhibit the expression of MBNL1 (Figure 6B‐E). Furthermore, trichostatin A (TSA) was used to block the function of p300. The results of Western blotting confirmed that ISO could not activate the expression of MBNL1 when the function of p300 was blocked (Figure 6F). The above results indicate that MBNL1 can be activated by MAPK and JNK pathways through p300.

### 3.7 Myocardin can reverse activate the transcription of MBNL1

**FIGURE 7:**
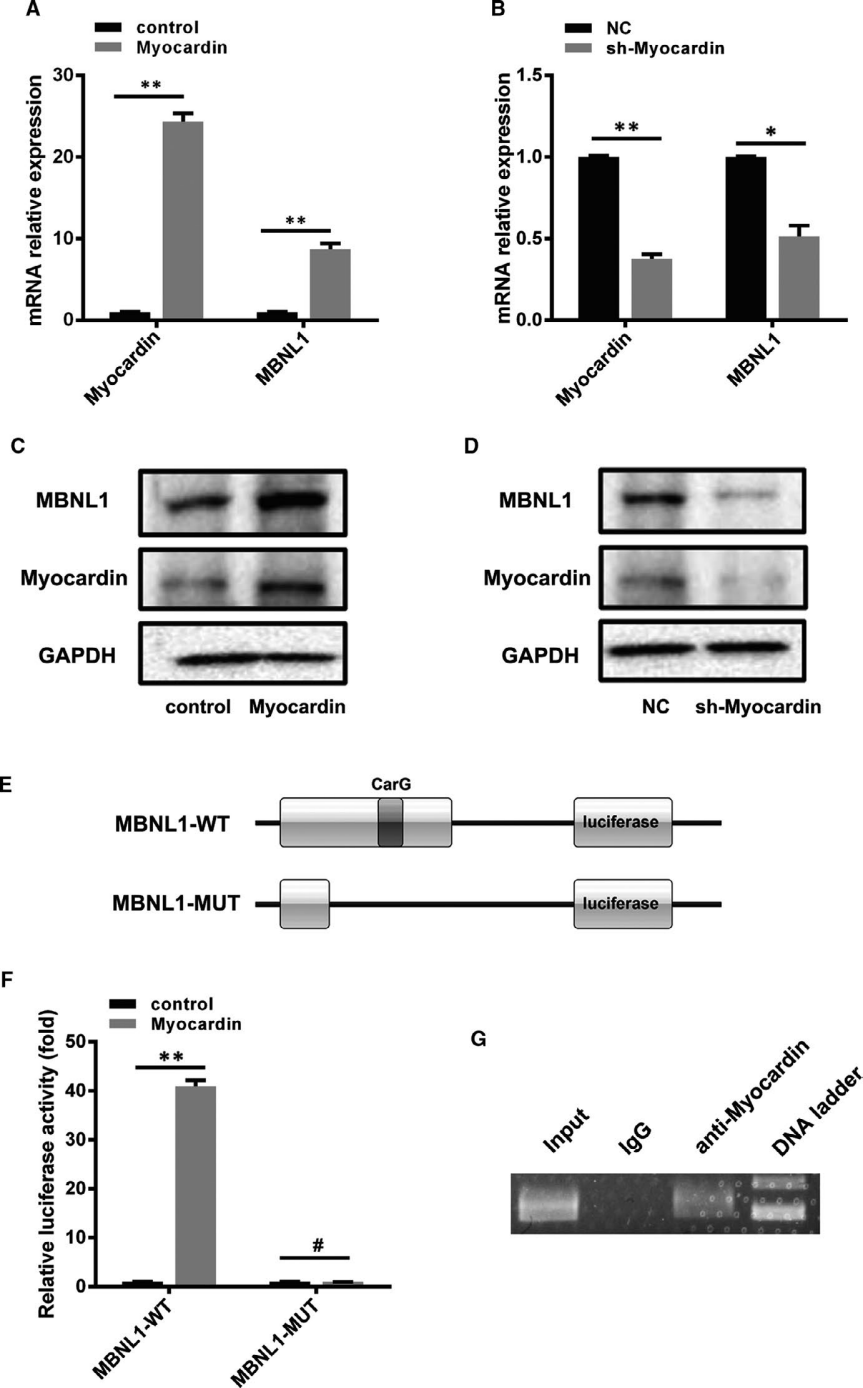
Myocardin can reverse activate the transcription of MBNL1. A‐D, Changes in MBNL1 and Myocardin in cardiomyocytes with overexpressed or silenced Mycoardin were detected using realtime PCR and Western blotting. (n = 3, *, *P* < .05, **, *P* < .01). E, MBNL1 promoter structure with or without the binding site (CarG box) of Mycoardin. F, The activation of Myocardin to wild‐type or mutant MBNL1 promoter was detected using luciferase assay. (n = 3, **, *P* < .01, ^#^, *P* > .05). G, The binding site of Myocardin was identified using ChIP assay

We analysed the promoter region of MBNL1 and found a Myocardin‐binding site CarG box. Does this mean that Myocardin can also regulate the expression of MBNL1? By using realtime PCR and Western blotting, we confirmed that Myocardin can significantly up‐regulate the expression of MBNL1 when Myocardin is overexpressed in cardiomyocytes; the opposite results were obtained after knocking down Myocardin (Figure 7A‐D). Then, we attempted to confirm the site of Myocardin acting on the MBNL1 promoter region using a luciferase assay. Our data indicated that Myocardin could significantly activate MBNL1 promoter transcription; however, when the CarG box was mutated, Myocardin could not activate MBNL1 promoter transcription (Figure 7E,F). This suggests that Myocardin can regulate the transcription and expression of MBNL1 through the CarG box. The above results were further confirmed by using chromatin immunoprecipitation (ChIP) assay (Figure 7G).

## 4 DISCUSSION

Hibernating myocardium has great effects on myocardial remodelling, which is caused by the decrease of coronary artery blood flow, leading to the continuous impairment of myocardial and left ventricular function. When the coronary artery blood supply improves or the myocardial oxygen consumption decreases, the cardiac function can be partially or completely restored to normal. Therefore, local microvascular reconstruction and functional recovery have become one of the important ways to reverse hibernating myocardium.^29^, ^30^ As a member of Myocardin family, MRTF‐A in endothelial cells can also promote the formation of local microvessels through SRF, partially improve hibernating myocardium status and regulate myocardial injury caused by myocardial ischaemia.^31^ Meanwhile, Mycoardin contributes to the differentiation of vascular smooth muscle.^32^ These reports suggest that Myocardin family may play a dual role in the process of myocardial remodelling. On the one hand, they can promote the process of myocardial remodelling such as myocardial hypertrophy; on the other hand, they can promote local angiogenesis, which is conducive to reversing hibernating myocardium. This may be a self‐protection of the body.

In this study, we confirmed that MBNL1 can increase the expression of Myocardin by inhibiting the degradation of Myocardin mRNA, which implicate myocardial hypertrophy and its subsequent pathophysiology. Myocardin is an important transcription factor in cardiovascular system development. The occurrence and development of myocardial hypertrophy are accompanied by the overexpression of Myocardin in cardiomyocytes. Previous studies have also confirmed that the forced overexpression of Myocardin in cardiomyocytes can induce myocardial hypertrophy.^5^ In this case, attempting to effectively control the over‐activation of Myocardin may be a potential method for blocking or delaying myocardial hypertrophy; therefore, elucidating the regulatory mechanism of Myocardin expression for the development of therapeutic strategies for myocardial hypertrophy.

Increasing evidence has demonstrated that a large number of factors have important effects on the expression or activity of Myocardin. The transcription factors Nkx2.5, KLF4 and TET2 can regulate the expression of Myocardin at the transcriptional level.^33^, ^34^, ^35^ P300 and GSK3 can acetylate and phosphorylate Myocardin protein, respectively.^36^, ^37^ At the post‐transcription level, a series of miRNAs have been reported to degrade Myocardin mRNA^38^, ^39^, ^40^; however, owing to the single function of miRNAs, they can only regulate the target gene by only binding to the 3'‐UTR. Moreover, whether other regulatory factors participate in the post‐transcriptional regulation of Myocardin is unclear. In fact, the post‐transcriptional regulatory mechanism includes multiple aspects: mRNA splicing, mRNA stability, mRNA localization and translation efficiency; therefore, exploring the post‐transcriptional regulation molecular network of Myocardin is necessary.

In response to environmental changes, the activation of intracellular signalling pathways results in upstream to downstream signalling, resulting in changes in the expression of certain genes, which in turn further lead to physiological or pathological changes such that adaptation to external pressure is achieved. In terms of a single gene, its expression is regulated at multiple levels, including transcription, post‐transcription, translation and apparent modification. Among them, the post‐transcriptional regulation is particularly delicate. During adaptive responses to environmental changes, the post‐transcriptional regulatory mechanism plays an important role. Various factors can be involved in post‐transcriptional regulation processes; these factors include lncRNAs, miRNAs and RBPs (RNA‐binding proteins). RBPs account for 6%‐8% of all proteins encoded and can control the fate of RNAs (maturation, transport, location and translation). Despite the importance of the functions of RBPs, only a very small number of RBPs have been identified, especially in diseases.

In recent years, many studies have focused on the involvement of RBPs in cardiac remodelling. The inhibition of RBM20 (RNA‐binding motif protein 20) expression leads to dilated cardiomyopathy (DCM) in adults.^41^, ^42^, ^43^ Moreover, PCBP2 can inhibit Ang II‐induced hypertrophy of cardiomyocytes by promoting GPR56 mRNA degradation.^44^ Here, we confirmed that MBNL1 directly binds to Myocardin mRNA to inhibit the degradation of Myocardin mRNA, thereby regulating the occurrence and development of myocardial hypertrophy. Our findings reveal a new function of MBNL1 in cardiac hypertrophy and the molecular network of the post‐transcriptional regulation of Myocardin.

In healthy hearts, fibroblasts play an important role in maintaining normal structure, electrical conduction, mechanical contraction and diastolic function. In main acting cells at pathological state, fibroblasts gather in the heart through various sources and pathways, and transform into myofibroblasts. The secretion of a large number of collagen and bioactive factors implicates myocardial fibrosis and provides a pathological basis for the occurrence and development of the disease. Previous reports have confirmed that MBNL1 can repair myocardial injury by up‐regulating the expression of SRF and promote the occurrence of fibrosis in the case of myocardial injury.^45^ Excessive fibrosis and cardiac hypertrophy may lead to pathological myocardial remodelling and even heart failure. In addition, human heart failure has been associated with reduced cardiac Nav1.5 Na^+^ channel current and SCN5A mRNA abundance.^46^ Studies have found that MBNL1 can regulate the splicing of SCN5A mRNA; such regulation lead to heart conduction defects in DM.^47^ These studies suggest that MBNL1 may play a regulatory role in myocardial hypertrophy, as we also have confirmed in this study.

Moreover, myocardial remodelling can cause cardiomyocyte apoptosis through the production of TNF‐α in large amounts and lead to heart failure.^48^ Here, we found that cardiomyocytes with overexpressed MBNL1 can produce more TNF‐α than normal after treatment with ISO. The prolonged presence of a large amount of TNF‐α in the microenvironment of cardiomyocytes will damage the cells. This suggests that the sustained high concentration of MBNL1 may contribute to heart failure via Myocardin and TNF‐α. Certainly, TNF‐α is not the only regulator of cardiomyocyte apoptosis. In fact, the mechanism of ISO‐induced cardiomyocyte apoptosis with MBNL1 overexpression is relatively complex; we will continue to study it in depth.

With the development of antisense oligonucleotide (ASO) technology, the expression or activity of some genes can be controlled. Our results suggest that MBNL1 may be a potential target for the treatment of myocardial remodelling. If ASOs or other small molecular inhibitors are designed for the combination of MBNL1/Myocardin and MBNL1/TNF‐α, it may be possible to inhibit cardiac hypertrophy and prevent the apoptosis of cardiomyocytes.

Multiple intracellular signalling pathways, including MAPK, JNK, PKC and PI3K, are important factors in myocardial remodelling.^49^, ^50^, ^51^, ^52^ We speculate that MBNL1 activation is related to these pathways in myocardial remodelling, and our results indicated that ISO could not activate MBNL1 when MAPK and JNK pathways were inhibited. Furthermore, we hope to identify the key points of MAPK and JNK in regulating MBNL1. As previously reported, activated MAPK can significantly up‐regulate the activity and expression of histone acetyltransferase p300.^53^ Meanwhile, p300 can also be regulated by the JNK pathway.^54^ As a histone acetyltransferase, p300 can regulate the expression of various target genes; however, the effect of p300 on MBNL1 expression is unclear. Here, we demonstrated that p300 can up‐regulate the expression of MBNL1. These results suggest that the MAPK and JNK pathways may activate the expression of MBNL1 via p300.

In this study, we explored the relationship between MBNL1 abundance in peripheral blood and cardiac hypertrophy which may provide a new direction for the early diagnosis of myocardial hypertrophy in the future. Myocardial remodelling is the result of multiple factors, including inflammatory factors, macrophages and so on. Among them, macrophage depletion can increase myocardial injury and accelerate the process of myocardial remodelling.^55^, ^56^, ^57^ Meanwhile, the high expression of MBNL1 in monocytes can inhibit the differentiation of macrophages.^58^ According to the above contents, we should detect the difference of MBNL1 expression in monocytes in myocardial remodelling model and normal mice to explore the potential of MBNL1 as a clinical diagnostic biomarker. However, we have explored the expression of MBNL1 in whole blood, which is more suitable for clinical diagnosis in the future than detecting the expression of MBNL1 in monocytes because of its convenience.

Interestingly, we found that Myocardin can reverse activate the transcription of MBNL1. When cardiac hypertrophy occurred, the overexpression of Myocardin also increased MBNL1 protein levels, which further strengthened the stability of Myocardin mRNA; this in turn maintained the increased abundance of Mycoardin and promoted myocardial remodelling.

Our study revealed the effect and molecular mechanism of MBNL1 in myocardial remodelling. Specifically, under the effects of hypertrophy stimulators such as ISO, MBNL1 can be activated by MAPK and JNK via p300. MBNL1 can up‐regulate the stability of Myocardin mRNA, resulting in the promotion of myocardial hypertrophy and myocardial fibrosis. Mycoardin can reverse activate the expression of MBNL1 and further accelerate myocardial remodelling. Meanwhile, the sustained activation of MBNL1 can induce cardiomyocytes to secrete TNF‐α and promote the apoptosis of cardiomyocytes (Figure S1). These findings not only provide molecular targets for the diagnosis and treatment of myocardial hypertrophy but also provide a theoretical basis for basic clinical research. Unfortunately, limited by experimental conditions, functional assessment such as in vivo ultrasound parameter tracking was lacking in this study.

## CONFLICT OF INTEREST

The authors declare that they have no conflict of interest.

## AUTHOR CONTRIBUTION

**Yao Xu:** Conceptualization (lead); Data curation (lead); Formal analysis (lead); Investigation (lead); Project administration (equal); Resources (lead); Supervision (equal); Validation (lead); Visualization (equal); Writing‐original draft (lead). **Chen Liang:** Software (supporting); Validation (supporting); Visualization (supporting). **Ying Luo:** Data curation (supporting); Formal analysis (supporting); Supervision (supporting); Validation (supporting); Writing‐original draft (supporting). **Tongcun Zhang:** Conceptualization (equal); Funding acquisition (lead); Project administration (equal); Supervision (equal).

## Supporting information

Fig S1
