# Supplementary figures and images for: MBNL1 regulates isoproterenol‐induced myocardial remodelling in vitro and in vivo
Source: J Cell Mol Med. 2020 Dec 8;25(2):1100–15. doi: 10.1111/jcmm.16177 (PMC7812249; doi:10.1111/jcmm.16177)

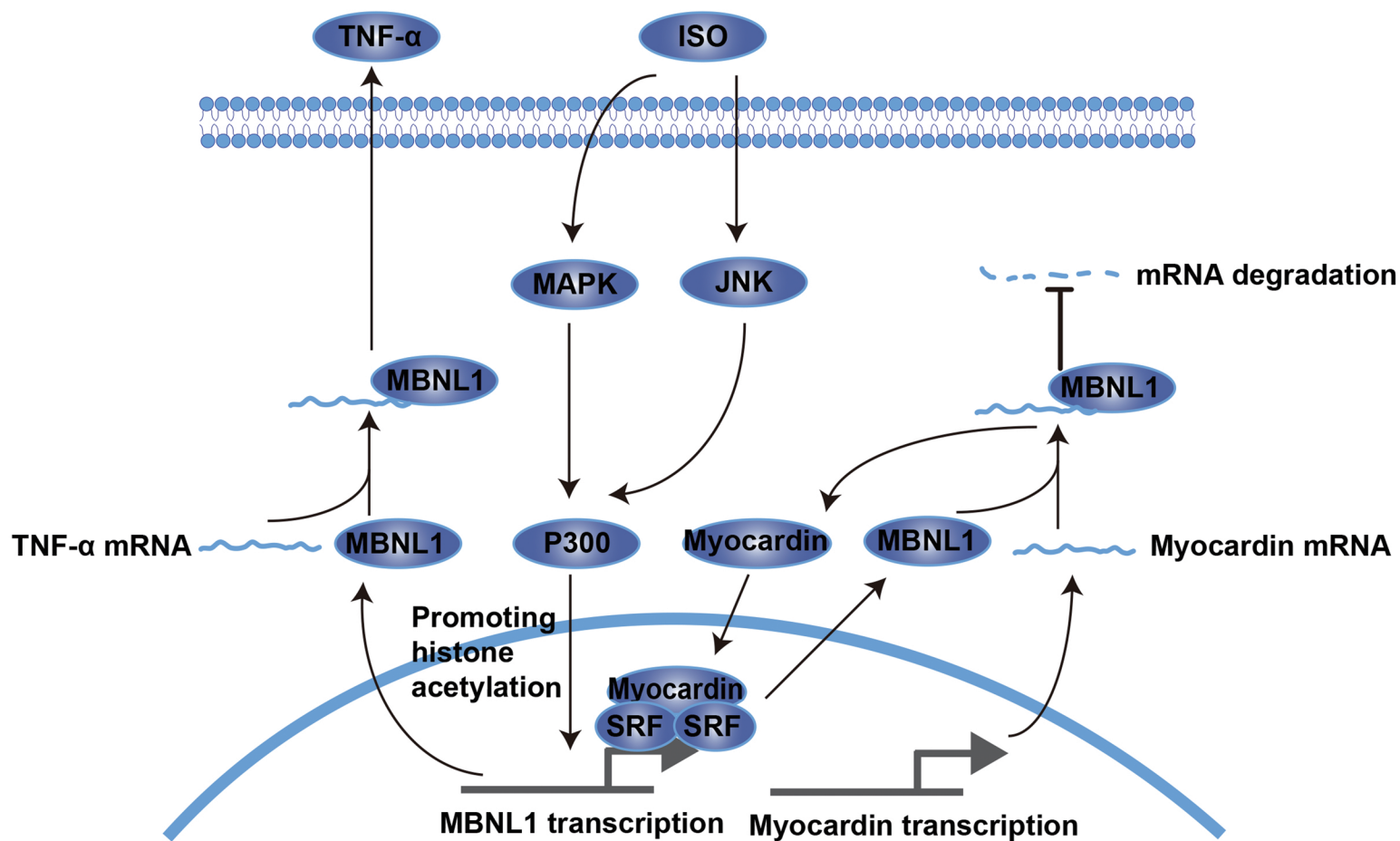

Supplementary figure 1

Supplement: Supplementary file 1 — Fig S1 [file JCMM-25-1100-s001.pdf]
